# Genome-wide assessment of genetic diversity and population structure of *Capsicum* in Southwestern Colombia using DArTseq markers

**DOI:** 10.1371/journal.pone.0359207

**Published:** 2026-09-25

**Authors:** Ronald Andrés Viáfara-Vega, Heiber Cárdenas-Henao

**Affiliations:** Grupo de Estudios Ecogenéticos y de Biología Molecular (ECOGENÉTICA), Facultad de Ciencias Naturales y Exactas, Universidad del Valle, Cali, Colombia; Birla Institute of Technology and Science, INDIA

## Abstract

Chili peppers (*Capsicum* spp.) are among the most widely cultivated horticultural crops worldwide due to the high demand for their pungent fruits. Despite Colombia’s high diversity of *Capsicum* species and varieties, its genetic resources remain underrepresented in international markets and scientific studies. This study aims to characterize the genetic diversity and population structure of *Capsicum* accessions from southwestern Colombia. Accessions were collected through field surveys in local markets and protected natural areas and genotyped using DArTseq platform. A total of 69 accessions representing seven species were genotyped using 3681 high quality DArT-SNP markers. Standard genetic diversity metrics and population structure analyses were performed. The analysis revealed low observed heterozygosity (mean 1%) and high inbreeding coefficients (F_IS_: 0.24). However, expected heterozygosity values indicate that these accessions still retain substantial genetic variability, highlighting their potential for breeding and genetic improvement programs. Population structure analysis revealed clear genetic differentiation between Andean and domesticated *Capsicum* species. Moreover, Silico-DArT markers could discriminate between wild and domesticated accessions within *C*. *annuum* and *C*. *frutescens*. This study represents the first genome wide assessments of Andean *Capsicum* species using DArTseq genotyping. These findings contribute to the understanding and conservation of *Capsicum* genetic resources of Southwestern Colombian and provide valuable information for future breeding programs.

## Introduction

The chili pepper belonging to the genus *Capsicum* of the Solanaceae family is among the eight most widely cultivated crops worldwide. In 2023, approximately 18.25 million hectares were cultivated, and a total production of 41.13 million tons was produced [1]. The high demand for chili peppers is due to their pungency, a characteristic unique to the genus caused by capsaicinoid compounds present in the placenta of the fruit [2]. Eleven species of *Capsicum* have been recorded in Colombia [3–5], five of which belong to the Andean clade. This group is distributed in the west-northwest of South America and Central America. The Andean clade is a distinct group from the other *Capsicum* species and differs from them in chemical, anatomical, and cytological characteristics [4,6,7]. For example, fruits of the Andean clade do not produce capsaicinoid compounds, lack giant mesocarp cells, and their karyotype number is 2n = 2x = 26. Despite their particularities, knowledge about their genetic diversity remains limited beyond the available systematic studies. In contrast, the domesticated species in Colombia belong to the *annuum* group. The most commonly cultivated species in Colombia are *C*. *annuum* L. (Cayenne variety), *C*. *frutescens* L. (Tabasco variety), and *C*. *chinense* Jacq. (Habanero variety). These varieties are cultivated in a wide range of climates throughout the country [8].

Despite its potential, hot pepper production in Colombia has not yet reached significant levels in the global context with an estimated annual production of approximately 100610 tons, the country ranks 32nd worldwide [1]. This limited participation could be explained by the narrow focus of studies conducted in the country. Although they have addressed the crop for agricultural and scientific purposes [9–12], they have focused on specific regions, and some do not include wild species. As a result, the diversity of chili peppers in Colombia is not well documented, representing a missed opportunity. This is especially relevant considering that Colombia includes two key centers of speciation of the *Capsicum* genus: the Andes and the Amazon [6]. Consequently, there is a valuable genetic resource that has not yet been comprehensively characterized, which limits its use in both agricultural research and development.

DNA markers have been widely used to analyze genetic diversity in groups of interest because they are not influenced by environmental factors and can be measured at any stage of an organism’s development [13]. Advances in sequencing technologies have enabled the use of a growing number of DNA markers, especially SNPs (single nucleotide polymorphisms), to cover a more extensive portion of the genome. This increased coverage provides more comprehensive information and enhanced utility [14,15].

Diversity Arrays Technology sequencing (DArTseq) platform [16] has established itself as an effective tool for analyzing plant genetic diversity. Its usefulness lies in the generation of a high density of markers, the absence of the need for prior sequence information, its broad genome coverage, and its ability to be transferred between species [17,18]. This technology produces two types of markers, dominant SilicoDArT markers, which indicate presence or absence, and codominant SNPs. Both have been successfully used to characterize genetic diversity in various plant species, such as rice [18,19], macadamia [20], bean [21], fava bean [22].

Worldwide, there are few studies evaluating the genetic diversity of chili pepper using *Capsicum* accessions from Colombia. In fact, many of these studies include limited or no representation of Colombian material [6,23–26]. When Colombian material is included, it typically comes from international germplasm banks that primarily conserve domesticated varieties, while wild or semi-domesticated accessions are rarely used. Given this situation, the need to conduct molecular evaluations that covers a representative part of the *Capsicum* genome becomes evident, as a basis for developing genetic improvement programs in Colombia aimed at strengthening production and increasing exports. This study is the first to apply the DArTseq platform to analyze *Capsicum* accessions from southwestern Colombia. It is also pioneering in the characterization of the genetic diversity of accessions belonging to the species of the Andean clade.

## Materials and methods

### Accessions sampling

Field trips were carried out in rural and urban areas of Valle del Cauca and Nariño departments to collect the studied species. Public markets, family gardens, and plant nurseries were visited to collect ripe fruits for seed extraction. To sample wild species, natural reserves and rural areas were visited. The people in charge of these places were asked about the origin of the material and, if it is known, the level of artificial selections to which the accessions were submitted. This verification process aimed to ensure that all collected samples were native and not introduced from other countries.

### Planting material and crop maintenance

A minimum of three individuals per accession were sown and cultivated under controlled conditions in a greenhouse at the biological station of the Universidad del Valle with geographic coordinates: 3º 22’22.23” N y 76º 31’47.82”O. Once the seedlings developed five leaves, foliar tissue was harvested and stored in silica gel bags to prevent DNA degradation, following the protocol described by Chase & Hills [27]. One individual per accession was used for genotyping.

### Samples identification

*Capsicum* accessions were identified through a combination of morphological traits including seed and flower color, annular constriction of calix, number of flowers per node [4] as well as DNA-HRM barcoding. The latter employed primers developed by Jeong *et al*. [28] to *Capsicum* species and subsequently validated for southwestern Colombian species [29].

### DNA extraction and genotyping

Genomic DNA was extracted from leaf tissue using the CTAB protocol described by Doyle & Doyle [30], with modifications according to Stewart [31]. DNA concentration and purity were assessed using Nanodrop 2000 spectrophotometer (Thermo Scientific, USA). Once quality parameters were confirmed, samples were submitted to the Genetic Analysis Service for Agriculture (SAGA) at CIMMYT (México) where genotyping process was performed using DArTseq™ technology [16,32]. Five accessions corresponding to commercial cultivars were included as control.

### Databases and data cleaning

This study examined seven species *of Capsicum*, including three wild Andean species (*C*. *lycianthoides* Bitter, *C*. *rhomboideum* (Dunal) Kuntze and *C*. *dimorphum* (Miers) Kuntze) and four domesticated (*C*. *annuum*, *C. frutescens*, *C. baccatum* var. *pendulum* (Willd.) Eshbaugh and *C. pubescens* Ruiz & Pav.). Due to significant morphological differences between Andean species and domesticated ones, there is a potential for bias in the molecular analysis. To address this, the molecular marker database was divided into two separate datasets, according to *Capsicum* species particularities.

The first dataset, referred to as the Total Database (BT), comprises all species collected in this study, both wild and domesticated. The second dataset, known as the Domesticated Database (BD), includes only domesticated species except *C*. *pubescens*, which was excluded due to its distinct morphological and molecular characteristics that make it easily identifiable [4]. Each dataset underwent independent data cleaning and genetic analysis.

Genotypic data were curated through five filters; all filters were made using DArTRverse package from R software. First, all monomorphic loci were filtered in the database. Then, loci with a reproducibility lower to 0.99 were filtered. For call rate filter, Due to the distinct genetic profile of Andean species compared to other *Capsicum* species analyzed in this study, some individuals from the Andean group exhibited missing data rate exceeding 10% when call rate filter of 0.89 was applied. These individuals were retained in the BT dataset, as understanding the relationship between Andean and domesticated groups were central objective of this study. However, for BD dataset a stricter call rate threshold of 95% was applied. The fourth filter eliminated secondaries loci, this consists in keeping only one locus in each DArT-seq fragment to reduce dependence between markers. For fifth filter, loci with MAF < 0.05 were filtered. Finally, an LD pruning was made for loci with a R^2^ > 0.2. Missing genotypes were imputed using the nearest-neighbor algorithm through gl.impute function of DArTRverse. The image in Supporting information 1 in S1 File summarizes the pipeline use in this study including the functions of DArTRverse.

### Genetic diversity and population structure analysis

SNP marker matrices and silico DArT were analyzed using DartRverse library [33,34] in the R environment, version 4.4.2 [35].. Using the functions of this library, diversity indices like H_o_, H_e_, and population structure statistics like Wrights’s F were estimated. For diversity indices, Andean species were excluded because of their low sample size. To visualize genetic relationships among *Capsicum* accessions, UPGMA dendrograms based on Euclidean distances (SNP-DArT) and Jaccard distances (Silico-DArT) were constructed, in both cases a bootstrap node support with 1000 replicates was made. In addition, the SNP markers were used population structure analysis using STRUCTURE v.2.3.4 [36], with the aim of estimating the number of subpopulations (K) present in the data. Ten individual Markov Chain Monte Carlo (MCMC) simulations were used for each K-value from 3 to 8 for BD and from 2 to 10 for BT. In both simulations, there was a burn-in phase of 100000 iterations, followed by 100000 iterations. The optimal number of subpopulations (k) was determined following the methodology proposed by [37] and the estimated log probability of the data, LnP(D), as implemented by [36]. Kindship matrix (k-matrix) and its heatmap were made using DArtRverse.

## Results

### Number of accessions collected

**Fig 1:**
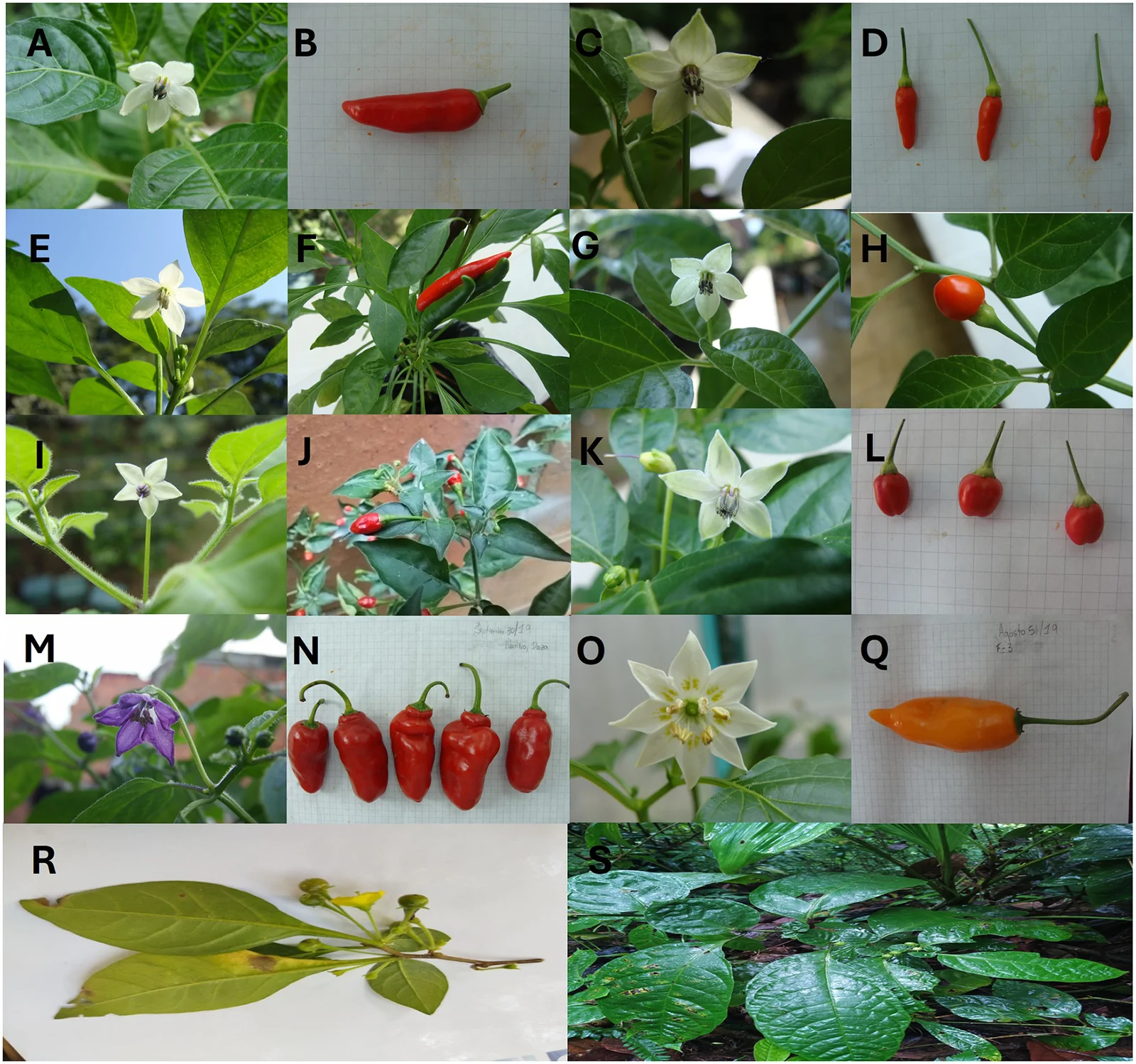
Flowers and fruits of *Capsicum* species collected in this study. a, b, e, f, i, j: *C*. *annuum*. c, d, g, h, k. l: *C*. *frutescens*. m-n: *C*. *pubescens*. o-q: *C*. *baccatum*. r: *C*. *rhomboideum*. s: *C*. *lycianthoides.*

A total of 69 accessions corresponding to seven *Capsicum* species were collected, as shown in Table 1 and Fig 1. The most represented species was *C*. *annuum,* with 38 accessions, followed by *C. frutescens* with 15. In the department of Valle del Cauca three wild *Capsicum* species belonging to the Andean clade were identified: *C*. *dimorphum* (one accession), *C*. *rhomboideum* (two accessions), and *C*. *lycianthoides* (three accessions). Of the total accessions, fifty-three were collected in the Valle del Cauca, and the remaining 16 in the department of Nariño. The species *C*. *pubescens* and *C*. *baccatum* were found exclusively in Nariño. The five commercial cultivars representing three species were included as controls: three samples of *C*. *annuum* var. annuum, one of *C*. *pubescens*, and one of *C*. *baccatum*.

**Table 1:** Number of *Capsicum* accessions and their distribution by department and species. *Caa*: *C. annuum* var. *annuum*, *Cag*: *C*. *annuum* var. *glabriusculum*, *Cfr*: *C*. *frutescens*, *Cba*: *C*. *baccatum*, *Cpu*: *C*. *pubescens, Cdi*: *C*. *dimorphum.*

Department	Species								Number of accessions
	*Caa*	*Cag*	*Cfr*	*Cpu*	*Cbp*	*Cdi*	*Crh*	*Cly*	
Nariño	2	2	1	6	5	0	0	0	16
Valle del Cauca	19	14	14	0	0	1	2	3	53
Total	21	16	15	6	5	1	2	3	69

### Data filtering

A total of 129810 SNP-DArT markers and 330763 Silico-DArT markers were genotyped using DArT-seq technology in 69 accessions. After the filtering process, the number of SNP markers was reduced to 3681 for BT and 2821 for BD, while the Silico-DArT markers were reduced to 140207 for BT and 2501 for BD. Furthermore, a total of 3258 SNP-DArT markers could be mapped on the twelve *Capsicum* chromosomes (Supporting information 2 in S1 File), demonstrating that the genomic reduction of the DArTseq library resulted in a nearly equal number of markers for each chromosome. The proportion of missing data prior to imputation was 7.72% in BT dataset and 1.66% in BD dataset. The slightly higher proportion of missing data in BT is likely attributable to the greater evolutionary divergence among the included species, which may reduce the recovery of homologous DArT-seq loci across taxa.

### Genetic diversity

SNP-DArT markers revealed a low level of observed heterozygosity in *Capsicum* accessions (Table 2). The heterozygosity ranges between 0.003 to 0.036, reaching a maximum of 0.187 in B6 (*C*. *annuum* accession). Similarly, expected heterozygosity was low, range between 0.003 to 0.040. There is a difference between two varieties of *C*. *annuum*, while *C*. *annuum* var. *annuum* showed the highest values as Ho as He, the wild variety *C*. *annuum* var. *glabriusculum* showed a low value. FIS indexes were variable, ranging from 0.007 to 0.330, suggesting an excess of homozygotes in *Capsicum* species. In the case of Andean accessions, the low sample size avoids inferring genetic diversity to species, but individuals showed a low H_o_ and H_e_ like other *Capsicum* species.

**Table 2:** Observed (H_O_) and expected Heterozygosity (uH_E_) and F_IS_ by *Capsicum* species.

Species	n	H_o_	uH_e_	FIS
*C. annuum var. annuum*	23	0.036 ± 0.024	0.040 ± 0.028	0.051 ± 0.222
*C. annuum var. glabriusculum*	16	0.006 ± 0.022	0.010 ± 0.033	0.252 ± 0.426
*C. baccatum*	5	0.021 ± 0.122	0.031 ± 0.118	0.330 ± 0.687
*C. pubescens*	6	0.014 ± 0.082	0.019 ± 0.082	0.317 ± 0.538
*C. frutescens*	13	0.003 ± 0.015	0.003 ± 0.017	0.007 ± 0.082

### Population structure

**Fig 2:**
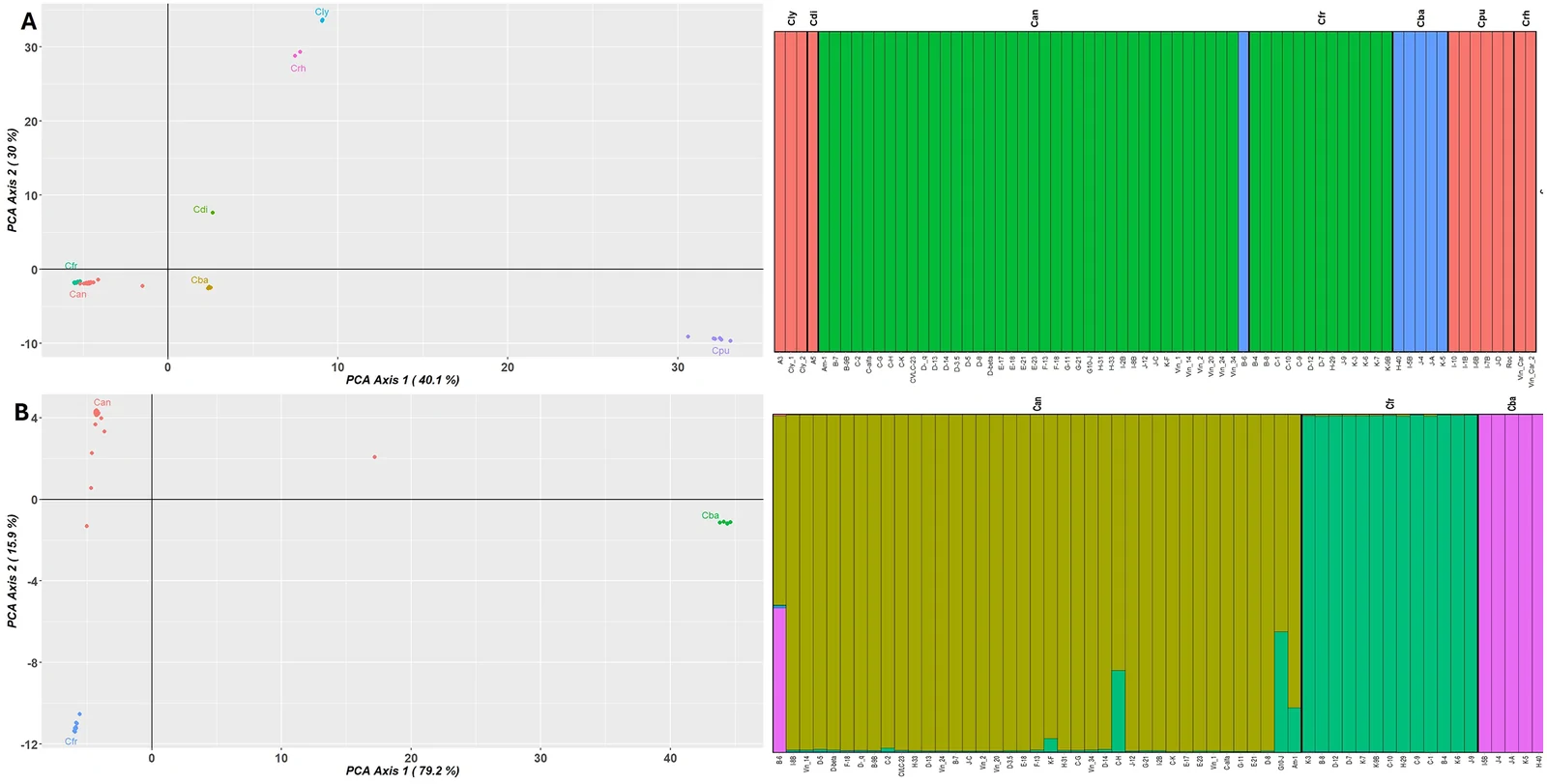
Principal component analysis and Structure plot using SNP for BT (file A) and BD (file B). *Can*: *C*. *annuum*, *Cbp*: *C*. *baccatum* var. *pendulum*, *Cdi*: *C*. *dimorphum*, *Cly*: *C*. *lycianthoides*, *Crh*: *C*. *rhomboideum*, *Cfr*: *C*. *frutescens* and *Cpu*: *C*. *pubescens*.

**Fig 3:**
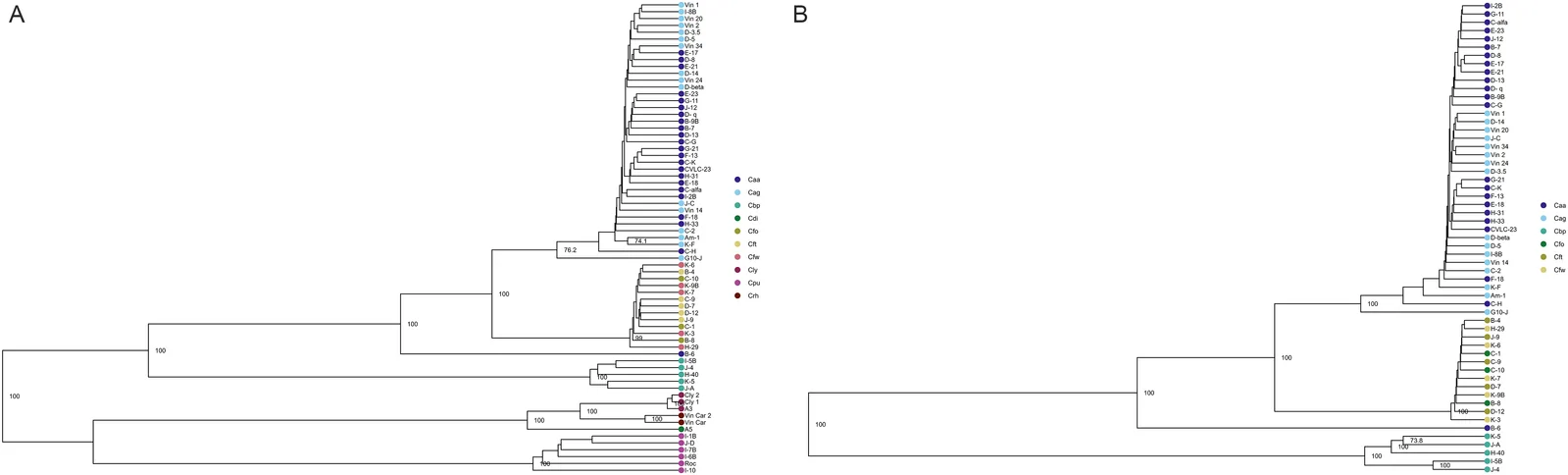
Dendrograms using Euclidean’s distance from SNP markers for BT (A) and BD (B) for *Capsicum* accessions. *Caa*: *C*. *annuum* var. *annuum*, *Cag*: *C*. *annuum* var. *glabriusculum*, *Cbp*: *C*. *baccatum* var. *pendulum*, *Cdi*: *C*. *dimorphum*, *Cly*: *C*. *lycianthoides*, *Crh*: *C*. *rhomboideum*, *Cft*: *C*. *frutescens* (tabasco variety), *Cfw*: *C*. *frutescens* (Wild variety), *Cfo*: *C*. *frutescens* (other varieties) and *Cpu*: *C*. *pubescens*. Bootstrap values greater than 70 are visible in each node.

The BT-based PCoA plots show a clear differentiation between Andean and domesticated accessions, both in the SNP analysis (Fig 2A) and in the Silico-DArT analysis (Fig 3A). The Andean group clustered separately of the rest of the accessions. Within the domesticated species, *C*. *pubescens* is the most separate group, while *C*. *annuum* and *C*. *frutescens* appear to be the closest species in this assessment. On the other hand, the BD-based PCoA plots (Fig 2B and 3B) show that excluding the Andean and *C*. *pubescens* species allows for a better understanding of the relationships between the accessions in the white-flower group. In this context, *C*. *baccatum* is positioned as the most distant species while, *C*. *annuum* and *C*. *frutescens* accessions were grouped as closest species but with no distinction among the varieties inside each species. F_ST_ pairwise index (Supporting information 3 in S1 File) reflects a similar structure for the *Capsicum* accessions with Andean accessions have the highest values of genetic differentiation against domesticated species, while *C*. *annuum* has the lowest value against *C*. *frutescens*.

STRUCTURE analysis suggested that the number of populations in the BT database is K = 3 (Supporting information 4 in S1 File). These three groups are Andean group and *C*. *pubescens*, *C*. *baccatum* and finally, *C*. *annuum* and *C*. *frutescens*. The assignment of accessions to the groups is 100% for all the cases. The relationship between Andean accessions, *C*. *pubescens* and the rest of the *Capsicum* species representing in this plot, agrees with the genetic divergence registered in PCoA plot. In fact, *C*. *pubescens* is so different to the rest of domesticated species that are clustered together with the Andean group. On the other hand, both the ΔK statistic and the plateau of the log-likelihood suggested K = 5 for BD as the optimal number of clusters, the STRUCTURE barplot revealed only three biologically meaningful genetic groups corresponding to *C. annuum*, *C. frutescens*, and *C. baccatum*. The remaining inferred clusters contributed negligibly to individual membership coefficients, indicating that additional clusters did not represent distinct genetic entities. This interpretation is further supported by the PCoA, UPGMA dendrogram, and kinship matrix, all of which consistently recovered the same three major genetic groups.

**Fig 4:**
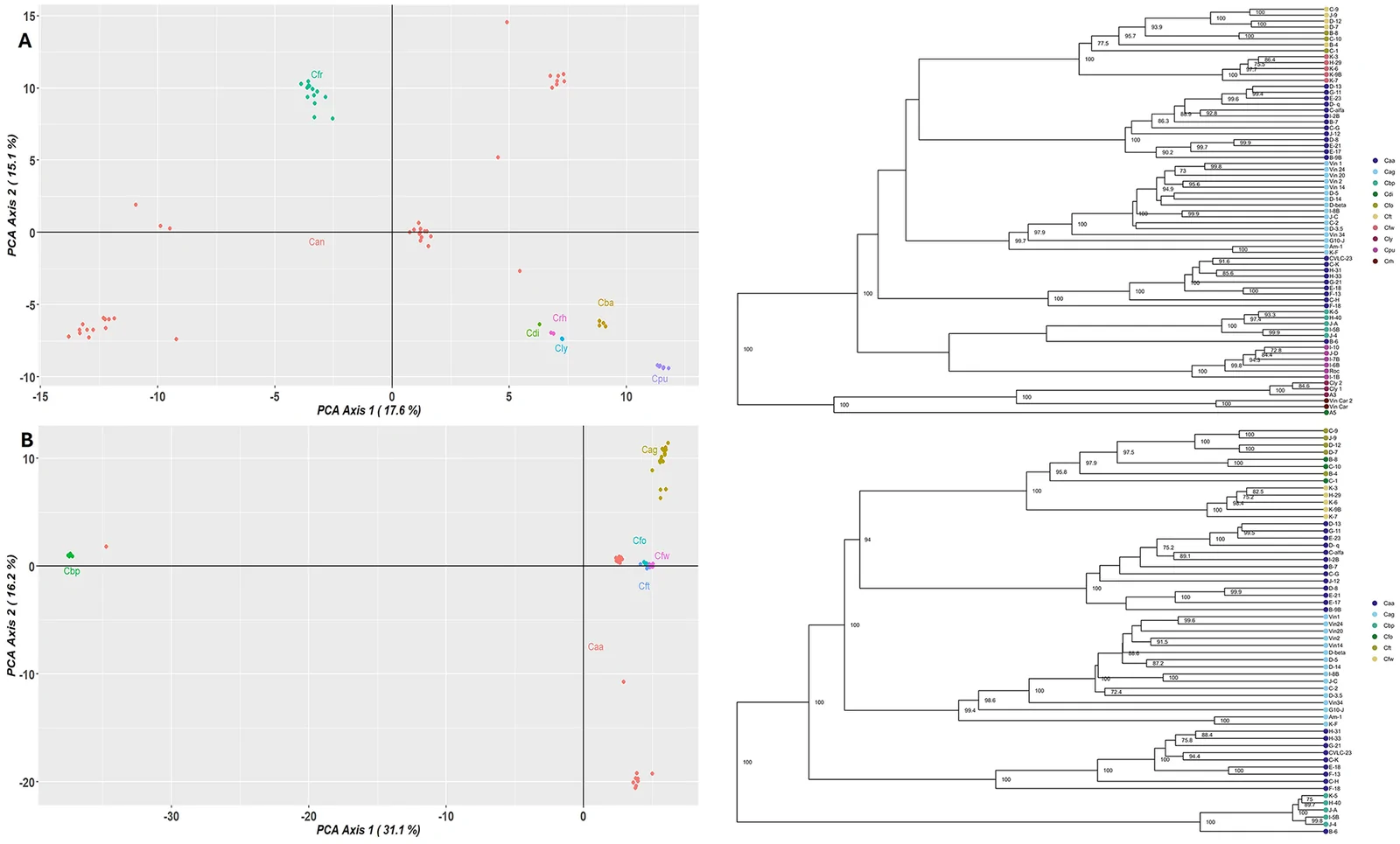
PCoA plot and UPGMA dendrograms using Jaccard’s distance from Silico-DArT markers for BT (A) and BD (B) for *Capsicum* accessions. *Caa*: *C*. *annuum* var. *annuum*, *Cag*: *C*. *annuum* var. *glabriusculum*, *Cbp*: *C*. *baccatum* var. *pendulum*, *Cdi*: *C*. *dimorphum*, *Cly*: *C*. *lycianthoides*, *Crh*: *C*. *rhomboideum*, *Cft*: *C*. *frutescens* (tabasco variety), *Cfw*: *C*. *frutescens* (Wild variety), *Cfo*: *C*. *frutescens* (other varieties) and *Cpu*: *C*. *pubescens*. Bootstrap values greater than 70 are visible in each node.

The UPGMA dendrograms constructed using Euclidean’s distance (Fig 3A) and Jaccard’s distance (Fig 4A) for BT show a high degree of concordance the PCoA plots. In both graphs, the Andean *Capsicum* accessions are grouped together and exhibit considerable distances from the domesticated species. Furthermore, accessions of *C*. *lycianthoides* and *C*. *rhomboideum* appear more closely related to each other than to *C*. *dimorphum* accession, a relationship also reflected in the PCoA results as SNP for Silico-DArT markers. Similarly, *C*. *pubescens* stands apart from the other domesticated species. Its clusters with the Andean group in the SNP-based dendrogram, while in the Silico-DArT dendrogram, its groups with the domesticated varieties. Regarding the white-flower group, both dendrograms display a consistent structure: *C*. *baccatum* forms the outer group, and *C*. *annuum* is separated from *C*. *frutescens*.

For BD, Euclidean’s distance and Jaccard’s distance agree to separate each domesticated species into a different cluster, with *C*. *baccatum* as outer group and *C*. *annuum* closer to *C*. *frutescens* as BT dendrograms. However, SNP and Silico-DArT markers reflect different clustering respect to varieties in *C*. *annuum* and *C*. *frutescens*. While SNP markers were unable to discriminate against varieties of both species with low values of boostrap in dendrogram. Silico-DArT was able to separate *C*. *annuum* into three well supported clusters, one of them corresponding to wild variety *C*. *annuum* var. *glabrisuculum* (Fig 4B). In the same way, *C*. *frutescens* wild variety was separated from the other accessions of this species with Silico-DArT.

**Fig 5:**
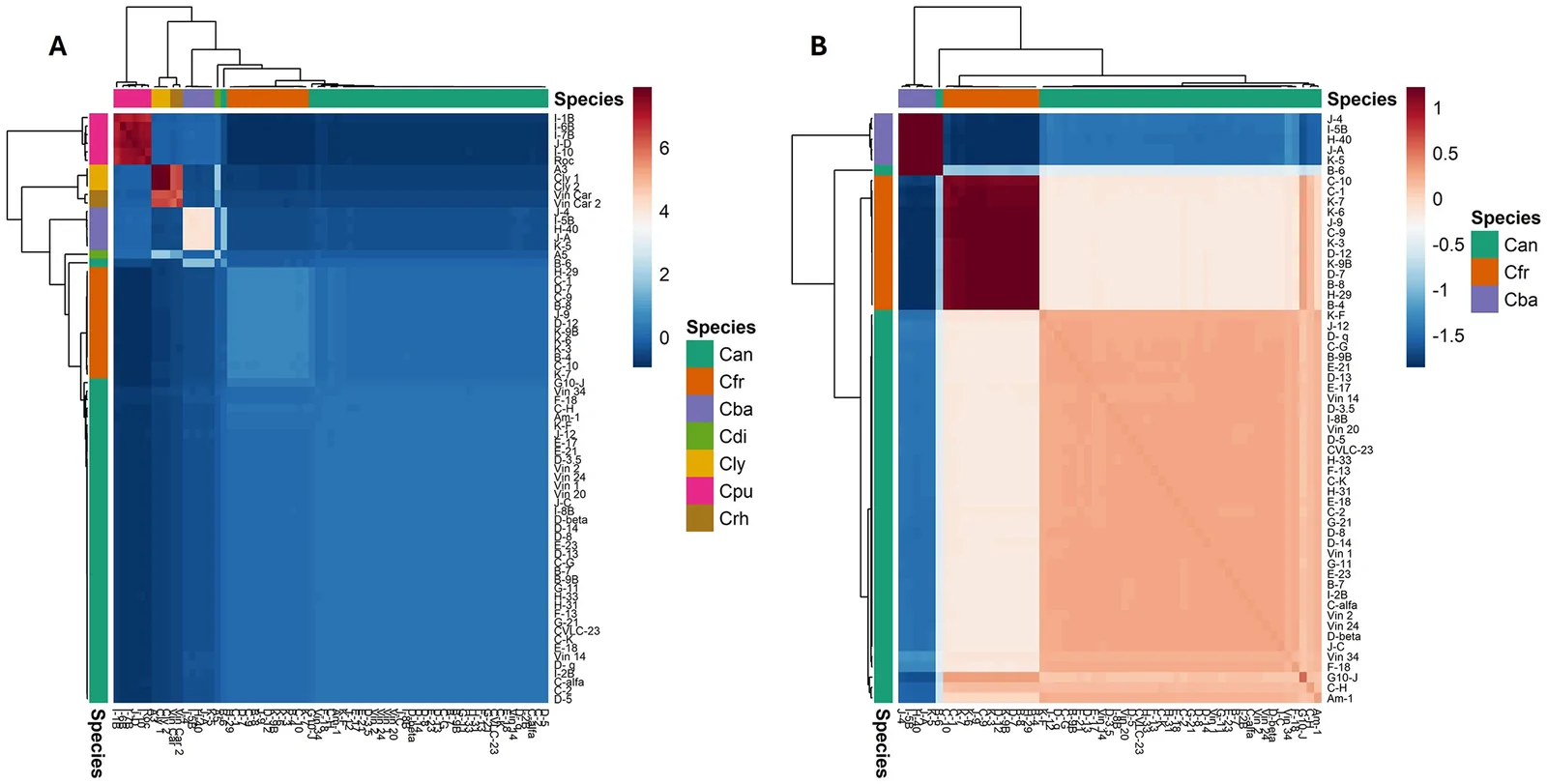
Heatmaps of the genomic kinship (K) matrices for the BT (A) and BD (B) datasets based on SNP markers. Hierarchical clustering was applied to group accessions according to their genomic relatedness. Colored side bars represent species assignment. Can: *C*. *annuum*, Cba: *C*. *baccatum* var. *pendulum*, Cdi: *C*. *dimorphum*, Cly: *C*. *lycianthoides*, Crh: *C*. *rhomboideum*, Cfr: *C*. *frutescens* and Cpu: *C*. *pubescens*.

Finally, the genomic kinship matrices for the BT(A) and BD(B) datasets based on SNP markers (Fig 5) revealed the same patterns of genetic differentiation that PCoA, STRUCTURE and distances dendrogram, with all accessions assigned to their respective species, showing the genetic divergence between domesticated *Capsicum* and Andean. B6 accession, despite the morphological identification assigned to *C*. *annuum*, showed different relationships depending on the analysis. For PCoA (SNP and Silico-DArT), STRUCTURE and Jaccard’s distances, this accession is group together *C*. *baccatum*, while Euclidean’s distances and K-matrix clustering as an outer group of the *C*. *annuum* and *C*. *frutescens* group. In both scenarios B6 is not cluster inside *C*. *annuum*, additionally, B6 was the accessions with the highest H_o_ value. Therefore, more analyses are necessary to solve its relationship in *Capsicum.*

## Discussion

### Patterns of genetic diversity and intraspecific differentiation

In this study, a low level of observed heterozygosity (H_O_) was observed, a recurring pattern in other *Capsicum* studies. For example, Ibiza *et al*. [38] reported a H_O_ value of 0.10 for *C*. *baccatum* and 0.05 for *annuum* complex and *C*. *pubescens* using AFLP markers. This pattern was also observed in studies using a large number of markers, for example, studies using GBS, such us [24,39,40] reported mean H_O_ of 0.023, 0.031 and 0.1, respectively. Notably, the last two studies reported a higher H_o_ in *C*. *baccatum* than in *annuum* complex species. Colonna *et al*. [26] is one of the few studies that found Ho higher than 0.3, which reported a Ho of 0.49 for a wild *Capsicum* species, *C*. *chacoense*, and found a lower value for domesticated species (*C*. *annuum* and *C*. *chinense*). For Colombian studies the pattern is similar, Pardey-Rodríguez and Dávila [11] and Viáfara *et al*. [41] reported a H_o_ of 0.1 and 0 for *Capsicum* accessions of *annuum* complex. The low H_o_ may be explained by the high level of autogamy and low level of autoincompatibility of the species of *Capsicum* genus [42–45], that tends to increase the homozygosity in the populations [46]. The high F_IS_ values found in this study show an excess of homozygotes in the accessions.

In this study, interspecific variation represented the dominant genetic signal and was therefore primarily recovered by the PCoA, STRUCTURE, and K-matrix analyses. Differences among varieties (intraspecific) represent a subtle signal, and it was recovered only by UPGMA/Jaccard analysis in this case. *C*. *annuum* and *C*. *frutescens* wild varieties are clearly distinguished from their domesticated counterparts using morphological characters (data not shown). The concordance between morphological differentiation and clustering of the UPGMA/Jaccard analysis suggests that these morphotypes probably reflect a real genetic structure though at a lower magnitude than the species differentiation. This intraspecific structure was also observed in other domesticated *Capsicum* species [47,48].

### Geographical structure and the role of public markets in maintaining *Capsicum* genetic diversity

This study shows an absence of geographical structure among accessions beyond that expected from species ecological preferences. *C*. *baccatum* and *C*. *pubescens* were found only in the Nariño department but this is explained by the fact that in this department the *Capsicum* cultivation occurs in the highlands (over 2000 m.a.s.l.) with low temperatures, these two species grow well in these conditions but *C*. *annuum* and *C*. *frutescens* are species more commonly cultivated in the lowlands with warmer temperatures, like those found in Valle del Cauca. Therefore, this geographical division is explained by the availability of suitable cultivation areas instead of an underlying genetic structure associated with geography.

The absence of geographical clustering suggests that in domesticated *Capsicum* species factors such as seed exchange, farmer’s selection and fruit trade could promote the dispersal of genetic material among multiple localities. For example, local farmers from different localities may select the same desirable traits of their plants, also, they could share seeds with their neighbors, these activities obscure detecting geographical structure, but may work to avoid local extinctions. This phenomenon has also been reported in other studies like in *C*. *annuum* var. *glabriusculum* in Mexico [49] or in domesticated *Capsicum* species [50].

The genetic diversity of *Capsicum* accessions is not only reflected in the results shown in this study but also in the number of collected accessions, the two departments sampled in this study represent less than 5% of the Colombian territory and it was found 69 *Capsicum* accessions. Many of these were collected from public markets instead of germplasm banks. Materials from different municipalities converge in these markets and they work as meeting point of *Capsicum* genetic resource. Interviews with farmers in public markets revealed that the fruits sold came from their own home gardens or farms located in the same or nearby veredas (rural administrative divisions), where they maintain a few *Capsicum* plants. Consequently, a public market acts as a meeting point for the genetic diversity of a microregion. Additionally, the presence of *Capsicum* accessions in public markets suggests that this resource is being used by the community and *Capsicum* fruits have economic value in a local context. Studies in Latin America, like Guatemala [51], Ecuador and Peru [52] and México [53,54] corroborate the importance of public markets in *Capsicum* genetic resources. Though they do not substitute germplasm banks could complement the strategies of prospection and conservation in situ of *Capsicum*.

### Genomic differentiation of the Andean *Capsicum* group

Andean group represents the ancestral group from the other *Capsicum* species; they have marked morphological and cytogenetic differences within which capsaicinoid absent and chromosomal number 2n = 2x = 26 are the most notorious [4]. Hence, it is coherent that genomic analysis reflects these differences, for example, PCoA, STRUCTURE analysis and Kinship matrix identified Andean accessions as a marked different cluster. Since this study is the first to includes Andean accessions in a genomic analysis of *Capsicum*, there is no studies to compare with, but, the genomic relationship within Andean group found in this study are same as the reported by phylogenetic analysis, with Andean accessions separated from the rest of *Capsicum* species and with *C*. *rhomboideum* more related with *C*. *lycianthoides* than with *C*. *dimorphum* [7]. H_o_ values for these six accessions didn’t surpass 1%, suggesting a high homozygotes level in these individuals, however further genetic studies into wild species of *Capsicum* are necessary to confirm the hypothesis of low level of heterozygosity in *Capsicum* Andean species.

In the STRUCTURE analysis and the UPGMA dendrogram with both types of markers, B6 was an accession that did not have a membership probability of 100%, indicating a relationship with *C*. *annuum* and *C*. *baccatum*. Morphologically, B6 has a white corolla without yellow spots and a calyx without conspicuous teeth, which are the main differences between *C*. *annuum* and *C*. *baccatum*, so a misidentification is unlikely. Furthermore, this accession exhibited the highest level of H_O_ in this study (0.187), a value forty-four times higher than the median and rare in other Colombian *Capsicum* studies, as previously mentioned. This suggests that B6 may represent a putative hybrid individual. However, additional analyses are required to confirm this hypothesis. This event could be favored by the traditional cultivation system, in which plants are kept by local farmers without physical isolation or pollination control [45]. To determine its agronomic potential, further studies on this accession are necessary.

### SNP-DArT vs Silico-DArT markers

In this study both types of markers used in genomic characterization showed similar results, as PCoA and as dendrograms could discriminate *Capsicum* accessions for species. Additionally, both types of markers recognized that Andean accessions have a big genetic divergence respect the other *Capsicum* accessions and *C*. *pubescens* is genetically different from the rest of domesticated species. Even B6 accession, was identified as problematic sample with both types of markers. This consistency in results obtained of these two markers have been reported by other studies that use DArT-seq platform [20,55].

The Silico-DArT UPGMA dendrogram resolved genetic subdivisions within *C*. *annuum* and *C*. *frutescens* that were not recovered by the SNP-based analyses. Because different marker systems and genetic distance measures were used, it is not possible to attribute this increased resolution exclusively to the marker type [56,57]. Nevertheless, the results suggest that fine-scale genetic structure within domesticated species exists but is likely masked by the much stronger interspecific differentiation in multivariate and model-based analyses.

Finally, DArT-seq technology allowed us to extract a good number of markers (SNP and Silico) in the *Capsicum* species evaluated in this study. Despite the great genetic differences between Andean group and domesticated species, 3681 SNP could be used to characterize the genetic diversity of these accessions. This suggested that DArT markers could be a good choice to execute genomic studies of various species within a genus. With respect to Silico-DArT, it is clear that the number of markers obtained is bigger than SNP and many of the analyses showed the same results than the SNP markers, however, do not allow the calculation of observed heterozygosity, hence its dominant characteristic and their great abundance make them more appropriate to genetic mapping, phenotypic association (GWAS) and plant identification.

### Limitations and perspectives

The region sampled in this study represent approximately 5% of Colombia’s geographic area. Therefore, the results of this study only represent a part of *Capsicum* genetic resources in the country. Despite this, the accessions showed a broad genetic diversity and multiples genetic groups. Though morphological diversity was not evaluated in this study, the accessions studied also display a broad morphological diversity. Hence, it is reasonable to think that expanding the sampling areas to other regions of the country would probably increase the genetic diversity and the number of genetic groups. Since most studies in Colombia have been focused on Amazonian and southwestern regions, studies focused on the Caribbean and Central region are necessary to adequately represent national diversity. In the same way, the results of this study justify the importance of amply national collections of *Capsicum* to conserve not only the wild species but also the diversity inside *C*. *annuum* and *C*. *frutescens*.

This study is the first to include Andean *Capsicum* species in a genomic characterization. However, the low and unbalanced sample size limits the ability to make important inferences. Unlike domesticated species, wild species are not abundant and finding individuals with flowers and/or fruits for an accurate taxonomic identification could be difficult. Therefore, this study presents an exploratory analysis of these species, a larger sample size is needed to confirm whether these groups also present a low level of H_o_ as it was observed in the domesticated species. Nevertheless, the differences in morphological and cytogenetic features reported by other studies [4,58] were also observed in this study using genomic analyses, the Andean accessions evaluated had a marked genetic divergence from the other accessions, even affecting the number of markers retained in the BT and BD databases, since Andean accessions shared fewer markers with domesticated accessions than domesticated accessions shared among themselves.

## Conclusions

This study reported the rich genetic diversity of *Capsicum* species distributed in Colombian southwest including some accessions that have been adapted to urban environments. Despite the unbalanced sample size, the dataset includes representatives of seven *Capsicum* species, including rarely studied Andean taxa. The use of DArT-seq markers reflects the genetic differentiation between Andean and domesticated *Capsicum* accessions. Moreover, they demonstrated that both groups have low levels of observed heterozygosity and a high level of inbreeding. However, there are groups genetically diverse and structured, suggesting a promising potential genetic resource for future breeding programs. Therefore, more studies with greater geographical coverage, bigger sample size and including other Andean *Capsicum* species are needed. In the same way, wild varieties inside *C*. *annuum* and *C*. *frutescens* could be discriminated. Overall, the DArT-seq platform proved to be a valuable tool to obtain a high number of markers in *Capsicum* species independently of its level of breeding and phylogenetic relationship.

## Supporting information

S1 File S1 Figure: Pipeline used for Quality control and genomic analyses. S2 Table: Chromosomal location of DArT-SNP markers. S3 Figure: F_ST_ index for *Capsicum* species. S4 Figure: STRUCTURE model selection plots (BT) showing mean LnP(K) and ΔK values used to infer the most likely number of genetic clusters. S5 Figure: STRUCTURE model selection plots (BD) showing mean LnP(K) and ΔK values used to infer the most likely number of genetic clusters.
(RAR)

## References

[1] FAOSTAT. Statistics of FAO. Rome: FAO; 2025 [cited 2025 Jul 14]. Available from: http://www.fao.org/faostat/es/#data/QC

[2] Palma JM, Terán F, Contreras-Ruiz A, Rodríguez-Ruiz M, Corpas FJ. Antioxidant profile of pepper (*Capsicum annuum* L.) fruits containing diverse levels of capsaicinoids. Antioxidants. 2020;9(9):878. doi: 10.3390/antiox9090878 PMC7554748 32957493

[3] Sua TSM, González MF, Cárdenas DL, Mendoza H. Base de datos de flora de la cuenca del río Orinoco en Colombia. Bogotá: Instituto Amazónico de Investigaciones Científicas Sinchi; Instituto de Investigación de Recursos Biológicos Alexander von Humboldt; 2017. http://ipt.biodiversidad.co/sinchi/resource?r=flora_orinoco&v=2.7

[4] Barboza GE, García CC, Bianchetti L de B, Romero MV, Scaldaferro M. Monograph of wild and cultivated chili peppers (Capsicum L., Solanaceae). PhytoKeys. 2022;200:1–423. doi: 10.3897/phytokeys.200.71667 36762372 PMC9881532

[5] Raz L, Agudelo Zamora HD. Herbario Nacional Colombiano (COL). Bogotá: Universidad Nacional de Colombia. 2023. doi: 10.15472/ea8sek

[6] Carrizo-García C, Barfuss MHJ, Sehr EM, Barboza GE, Samuel R, Moscone EA. Phylogenetic relationships, diversification and expansion of chili peppers (*Capsicum*, Solanaceae). Ann Bot. 2016;118(1):35–51. 10.1093/aob/mcw079 PMC4934398 27245634

[7] Barboza GE, Carrizo García C, Leiva González S, Scaldaferro M, Reyes X. Four new species of *Capsicum* (Solanaceae) from the tropical Andes and an update on the phylogeny of the genus. PLoS ONE. 2019;14(1):e0209792. doi: 10.1371/journal.pone.0209792 PMC6334993 30650102

[8] Bonilla-Granja MB, Viáfara-Vega RA, Cárdenas-Henao H. Ajíes del suroccidente colombiano. In: Katz E, Vásquez-Dávila MA, Aguilar-Meléndez A, Manzanero-Medina GI. Chiles, ajíes y pimentas: Capsicum en el Caribe, Centro y Sudamérica. Veracruz: Universidad Veracruzana; 2021. p. 259–72.

[9] Gómez MSH, García JAB, Melgarejo LM, Bardales X. Caracterización y usos potenciales del banco de germoplasma de ají amazónico. Bogotá: Instituto Amazónico de Investigaciones Científicas SINCHI; 2004.

[10] Medina CI, Lobo M, Gómez AF. Variabilidad fenotípica en poblaciones de ají y pimentón de la colección colombiana del género Capsicum. CTA. 2007;7(2):25–39. doi: 10.21930/rcta.vol7_num2_art:67

[11] Pardey-Rodríguez CP, Dávila MAG. Caracterización molecular de 135 introducciones de Capsicum procedentes del banco de germoplasma de la Universidad Nacional de Colombia sede Palmira. Intropica. 2011;6(1):21–32.

[12] Villota-Cerón D, Bonilla-Betancourt ML, Carmen-Carrillo H, Jaramillo-Vásquez J, García-Dávila MA. Caracterización morfológica de introducciones de Capsicum spp. existentes en el banco de germoplasma activo de Corpoica CI Palmira, Colombia. Acta Agron. 2012;61(1):16–26.

[13] Bolibok-Brągoszewska H, Rakoczy-Trojanowska M. Molecular marker-based assessment of genetic diversity in rye. Genetic diversity and erosion in plants: indicators and prevention. Cham: Springer International Publishing; 2015. p. 105–23.

[14] Andrews KR, Good JM, Miller MR, Luikart G, Hohenlohe PA. Harnessing the power of RADseq for ecological and evolutionary genomics. Nat Rev Genet. 2016;17(2):81–92. doi: 10.1038/nrg.2015.28 PMC4823021 26729255

[15] Allan V, Vetriventhan M, Senthil R, Geetha S, Deshpande S, Rathore A, et al. Genome-Wide DArTSeq Genotyping and Phenotypic Based Assessment of Within and Among Accessions Diversity and Effective Sample Size in the Diverse Sorghum, Pearl Millet, and Pigeonpea Landraces. Front Plant Sci. 2020;11. doi: 10.3389/fpls.2020.587426 PMC7768014 33381130

[16] Sansaloni C, Petroli C, Jaccoud D, Carling J, Detering F, Grattapaglia D, et al. Diversity Arrays Technology (DArT) and next-generation sequencing combined: genome-wide, high throughput, highly informative genotyping for molecular breeding of Eucalyptus. BMC Proc. 2011;5(S7). doi: 10.1186/1753-6561-5-s7-p54

[17] Sansaloni CP, Petroli CD, Carling J, Hudson CJ, Steane DA, Myburg AA, et al. A high-density Diversity Arrays Technology (DArT) microarray for genome-wide genotyping in Eucalyptus. Plant Methods. 2010;6(1). doi: 10.1186/1746-4811-6-16 PMC2903579 20587069

[18] Thant AA, Zaw H, Kalousova M, Singh RK, Lojka B. Genetic Diversity and Population Structure of Myanmar Rice (Oryza sativa L.) Varieties Using DArTseq-Based SNP and SilicoDArT Markers. Plants. 2021;10(12):2564. doi: 10.3390/plants10122564 PMC8707408 34961035

[19] Ndjiondjop M-N, Semagn K, Gouda AC, Kpeki SB, Dro Tia D, Sow M, et al. Genetic Variation and Population Structure of Oryza glaberrima and Development of a Mini-Core Collection Using DArTseq. Front Plant Sci. 2017;8. doi: 10.3389/fpls.2017.01748 PMC5651524 29093721

[20] Alam M, Neal J, O’Connor K, Kilian A, Topp B. Ultra-high-throughput DArTseq-based silicoDArT and SNP markers for genomic studies in macadamia. PLoS ONE. 2018;13(8):e0203465. doi: 10.1371/journal.pone.0203465 PMC6118395 30169500

[21] Ketema S, Tesfaye B, Keneni G, Amsalu Fenta B, Assefa E, Greliche N, et al. DArTSeq SNP-based markers revealed high genetic diversity and structured population in Ethiopian cowpea [Vigna unguiculata (L.) Walp] germplasms. PLoS ONE. 2020;15(10):e0239122. doi: 10.1371/journal.pone.0239122 PMC7544073 33031381

[22] Ohm H, Åstrand J, Ceplitis A, Bengtsson D, Hammenhag C, Chawade A, et al. Novel SNP markers for flowering and seed quality traits in faba bean (Vicia faba L.): characterization and GWAS of a diversity panel. Front Plant Sci. 2024;15. doi: 10.3389/fpls.2024.1348014 PMC10950902 38510437

[23] Lee H-Y, Ro N-Y, Jeong H-J, Kwon J-K, Jo J, Ha Y, et al. Genetic diversity and population structure analysis to construct a core collection from a large Capsicum germplasm. BMC Genet. 2016;17(1). doi: 10.1186/s12863-016-0452-8 PMC5109817 27842492

[24] Taranto F, D’Agostino N, Greco B, Cardi T, Tripodi P. Genome-wide SNP discovery and population structure analysis in pepper (*Capsicum annuum*) using genotyping by sequencing. BMC Genomics. 2016;17(1). doi: 10.1186/s12864-016-3297-7 PMC5117568 27871227

[25] Tripodi P, Greco B. Large scale phenotyping provides insight into the diversity of vegetative and reproductive organs in a wide collection of wild and domesticated peppers (*Capsicu*m spp.). Plants. 2018;7(4):103. doi: 10.3390/plants7040103 PMC6313902 30463212

[26] Colonna V, D’Agostino N, Garrison E, Albrechtsen A, Meisner J, Facchiano A, et al. Genomic diversity and novel genome-wide association with fruit morphology in Capsicum, from 746k polymorphic sites. Sci Rep. 2019;9(1):1–14. 10.1038/s41598-019-46136-5 PMC6624249 31296904

[27] Chase MW, Hills HH. Silica gel: An ideal material for field preservation of leaf samples for DNA studies. TAXON. 1991;40(2):215–20. doi: 10.2307/1222975

[28] Jeong H-J, Jo YD, Park S-W, Kang B-C. Identification of *Capsicum* species using SNP markers based on high resolution melting analysis. Genome. 2010;53(12):1029–40. doi: 10.1139/G10-094 21164536

[29] Viáfara-Vega RA, Cárdenas-Henao H. Identification of *Capsicum* species from Colombia by DNA barcoding and high resolution melting (HRM) analysis. Genet Resour Crop Evol. 2024;72(2):2277–86. doi: 10.1007/s10722-024-02097-x

[30] Doyle JJ, Doyle JL. A rapid DNA isolation procedure for small quantities of fresh leaf tissue. Phytochem Bull. 1987;19:11–5.

[31] Stewart CN. Rapid DNA Extraction from Plants. Fingerprinting Methods Based on Arbitrarily Primed PCR. Springer Berlin Heidelber; 1997. p. 25–8. doi: 10.1007/978-3-642-60441-6_4

[32] Ren J, Sun D, Chen L, You FM, Wang J, Peng Y. Genetic diversity revealed by single nucleotide polymorphism markers in a worldwide germplasm collection of durum wheat. Int J Mol Sci. 2013;14(4):7061–88. 10.3390/ijms14047061 PMC3645677 23538839

[33] Gruber B, Unmack PJ, Berry OF, Georges A. dartr: An r package to facilitate analysis of SNP data generated from reduced representation genome sequencing. Mol Ecol Resour. 2018;18(3):691–9. doi: 10.1111/1755-0998.12745 29266847

[34] Mijangos JL, Gruber B, Berry O, Pacioni C, Georges A. dartR v2: An accessible genetic analysis platform for conservation, ecology and agriculture. Methods Ecol Evol. 2022;13(10):2150–8. doi: 10.1111/2041-210x.13918

[35] R Core Team. R: A language and environment for statistical computing [Internet]. Vienna: R Foundation for Statistical Computing; 2024. Available from: https://www.r-project.org/

[36] Pritchard JK, Stephens M, Donnelly P. Inference of population structure using multilocus genotype data. Genetics. 2000;155(2):945–59. doi: 10.1093/genetics/155.2.945 PMC1461096 10835412

[37] Evanno G, Regnaut S, Goudet J. Detecting the number of clusters of individuals using the software STRUCTURE: A simulation study. Molecular Ecology. 2005;14:2611–20. 10.1111/j.1365-294X.2005.02553.x 15969739

[38] Ibiza VP, Blanca J, Cañizares J, Nuez F. Taxonomy and genetic diversity of domesticated *Capsicum* species in the Andean region. Genet Resour Crop Evol. 2011;59(6):1077–88. doi: 10.1007/s10722-011-9744-z

[39] Pereira Dias L, Vilanova S, Fita A, Prohens J, Rodríguez-Burruezo A. Genetic diversity, population structure, and relationships in a collection of pepper (*Capsicum* spp.) landraces from the Spanish center of diversity revealed by genotyping by sequencing (GBS). Hortic Res. 2019;6. 10.1038/s41438-019-0132-8 PMC6491490 31044080

[40] Tripodi P. Genomic structure and marker-trait association for plant and fruit traits in *Capsicum chinense* and *Capsicum baccatum* germplasm. BMC Res Notes. 2024;17(1). doi: 10.1186/s13104-024-06889-3 PMC11337620 39169427

[41] Viáfara-Vega RA, Cifuentes HG, Cárdenas-Henao H. Molecular characterization of cultivars of *Capsicum annuum* L. and *Capsicum frutescen*s L. (Solanaceae) from Valle del Cauca, Colombia by SSR-HRM technique. Genet Resour Crop Evol. 2024;72(4):4539–50. doi: 10.1007/s10722-024-02219-5

[42] Odland ML, Porter AM. A study of natural crossing in pepper (*Capsicum frutescens* L.). J Am Soc Hortic Sci. 1941;38:585–8.

[43] Onus AN, Pickersgill B. Unilateral incompatibility in *Capsicum* (Solanaceae): occurrence and taxonomic distribution. Ann Bot. 2004;94(2):289–95. doi: 10.1093/aob/mch139 15229125 PMC4242164

[44] Justino EV, Fonseca MEN, Ferreira ME, Boiteux LS, Silva PP, Nascimento WM. Estimate of natural cross pollination rate of *Capsicum annuum* using a codominant molecular marker associated with fruit pungency. Genet Mol Res. 2018;17(1). doi: 10.4238/gmr16039887

[45] Parry C, Wang Y-W, Lin S, Barchenger DW. Reproductive compatibility in *Capsicum* is not necessarily reflected in genetic or phenotypic similarity between species complexes. PLoS ONE. 2021;16(3):e0243689. doi: 10.1371/journal.pone.0243689 PMC8508556 33760824

[46] Falconer DS, Mackay TFC. Introducción a la genética cuantitativa. 4th ed. Zaragoza: Editorial Acribia; 2006. p. 468.

[47] Albrecht E, Zhang D, Mays AD, Saftner RA, Stommel JR. Genetic diversity in Capsicum baccatumis significantly influenced by its ecogeographical distribution. BMC Genet. 2012;13(1). doi: 10.1186/1471-2156-13-68 PMC3496591 22866868

[48] González-Pérez S, Garcés-Claver A, Mallor C, Sáenz de Miera LE, Fayos O, Pomar F, et al. New Insights into *Capsicum* spp relatedness and the diversification process of *Capsicum annuum* in Spain. PLoS ONE. 2014;9(12):e116276. doi: 10.1371/journal.pone.0116276 PMC4278865 25545628

[49] González-Jara P, Moreno-Letelier A, Fraile A, Piñero D, García-Arenal F. Impact of human management on the genetic variation of wild pepper, Capsicum annuum var. glabriusculum. PLoS ONE. 2011;6(12):e28715. doi: 10.1371/journal.pone.0028715 PMC3232243 22163053

[50] Liu F, Zhao J, Sun H, Xiong C, Sun X, Wang X, et al. Genomes of cultivated and wild Capsicum species provide insights into pepper domestication and population differentiation. Nat Commun. 2023;14(1). doi: 10.1038/s41467-023-41251-4 PMC10484947 37679363

[51] Guzmán FA, Ayala H, Azurdia C, Duque MC, De Vicente MC. AFLP assessment of genetic diversity of Capsicum genetic resources in Guatemala: Home gardens as an option for conservation. Crop Science. 2005;45(1):363–70.

[52] van Zonneveld M, Ramirez M, Williams DE, Petz M, Meckelmann S, Avila T, et al. Screening genetic resources of *Capsicum* Peppers in their primary center of diversity in Bolivia and Peru. PLoS ONE. 2015;10(9):e0134663. doi: 10.1371/journal.pone.0134663 PMC4581705 26402618

[53] Aguilar-Meléndez A, Katz E, Vásquez-Dávila MA, Barboza GE. Capsicum annuum L. var. annuum, Capsicum annuum L. var. glabriusculum (Dunal) Heiser & Pickersgill, Capsicum chinense Jacq., Capsicum frutescens L., Capsicum lanceolatum (Greenm.) CV Morton & Standley, Capsicum pubescens Ruiz & Pav., Capsicum rhomboideum (Dunal) Kuntze (Solanaceae). Ethnobotany of the Mountain Regions of Mexico. Cham: Springer International Publishing; 2022. p. 1–17.

[54] Aguilar-Meléndez A, Morrell PL, Roose ML, Kim SC. Genetic diversity and structure in semiwild and domesticated chiles (*Capsicum annuum*; Solanaceae) from Mexico. Am J Bot. 2009;96(6):1190–202. 10.3732/ajb.0800155 21628269

[55] Baloch FS, Lee SM, Mansoor S, Morales A, Karunathilake EMBM, Nadeem MA, et al. GBS-derived SNP and SilicoDArT markers reveals the genetic variation and population structure of Korean buckwheat (*Fagopyrum esculentum*) an underutilised crop. BMC Plant Biol. 2025;25(1). doi: 10.1186/s12870-025-07633-0 PMC12574297 41168792

[56] Kosman E, Leonard KJ. Similarity coefficients for molecular markers in studies of genetic relationships between individuals for haploid, diploid, and polyploid species. Molecular Ecology. 2005;14(2):415–24. doi: 10.1111/j.1365-294x.2005.02416.x 15660934

[57] Bonin A, Ehrich D, Manel S. Statistical analysis of amplified fragment length polymorphism data: A toolbox for molecular ecologists and evolutionists. Molecular Ecology. 2007;16(18):3737–58. doi: 10.1111/j.1365-294x.2007.03435.x 17850542

[58] Moscone EA, Scaldaferro MA, Grabiele M, Cecchini NM, Sanchez García Y, Jarret R, et al. The evolution of chili peppers (Capsicum-Solanaceae): A cytogenetic perspective. In: VI International Solanaceae Conference: Genomics Meets Biodiversity, 2006. 137–70.

